# Row ratio increasing improved light distribution, photosynthetic characteristics, and yield of peanut in the maize and peanut strip intercropping system

**DOI:** 10.3389/fpls.2023.1135580

**Published:** 2023-07-14

**Authors:** Juntian Lu, Qiqi Dong, Guohu Lan, Zecheng He, Dongying Zhou, He Zhang, Xiaoguang Wang, Xibo Liu, Chunji Jiang, Zheng Zhang, Shubo Wan, Xinhua Zhao, Haiqiu Yu

**Affiliations:** ^1^ College of Agronomy, Shenyang Agricultural University, Shenyang, Liaoning, China; ^2^ Maize Research Institute, Dandong Academy of Agricultural Sciences, Dandong, Liaoning, China; ^3^ Key Laboratory of Crop Genetic Improvement, Ecology and Physiology, Shandong Academy of Agricultural Sciences, Jinan, Shandong, China

**Keywords:** maize and peanut intercropping, row ratio configurations, photosynthetically active radiation, chlorophyll synthesis, photosynthetic characteristics

## Abstract

Changes in the canopy microclimate in intercropping systems, particularly in the light environment, have important effects on the physiological characteristics of photosynthesis and yield of crops. Although different row ratio configurations and strip widths of dwarf crops in intercropping systems have important effects on canopy microclimate, little information is available on the effects of intercropping on chlorophyll synthesis and photosynthetic physiological properties of dwarf crops. A 2-year field experiment was conducted in 2019 and 2020, with five treatments: sole maize (SM), sole peanut (SP), four rows of maize intercropping with eight rows of peanut (M4P8), four rows of maize intercropping with four rows of peanut (M4P4), and four rows of maize intercropping with two rows of peanut (M4P2). The results showed that the light transmittance [photosynthetically active radiation (PAR)], photosynthetic rate (*Pn*), transpiration rate (*Tr*), and stomatal conductance (*Gs*) of intercropped peanut canopy were reduced, while the intercellular carbon dioxide concentration (*Ci*) was increased, compared with SP. In particular, the M4P8 pattern *Pn* (2-year mean) was reduced by 5.68%, 5.33%, and 5.30%; *Tr* was reduced by 7.41%, 5.45%, and 5.95%; and *Gs* was reduced by 8.20%, 6.88%, and 6.46%; and *Ci* increased by 11.95%, 8.06%, and 9.61% compared to SP, at the flowering needle stage, pod stage, and maturity, respectively. M4P8 improves the content of chlorophyll synthesis precursor and conversion efficiency, which promotes the utilization efficiency of light energy. However, it was significantly reduced in M4P2 and M4P4 treatment. The dry matter accumulation and pod yield of peanut in M4P8 treatment decreased, but the proportion of dry matter distribution in the late growth period was more transferred to pods. The full pod number decreases as the peanut row ratio decreases and increases with year, but there is no significant difference between years. M4P8 has the highest yield and land use efficiency and can be used as a reference row ratio configuration for maize–peanut intercropping to obtain relatively high yield benefits.

## 1 Introduction

Intercropping, as a temporally and spatially intensive cultivation technology model, is widely applied by farmers in modern agricultural production across the world due to the efficient utilization of natural resources, higher land equivalent ratio (LER), and ecological benefits (Yu et al., 2015; Raseduzzaman and Jensen, 2017; Nelson et al., 2018). In the reasonable intercropping systems, the high cereal crops intercropping with lower legume crops are usually used to improve the ventilation and light condition of cereal crops and increase nutrient use efficiency and yield (Zhang et al., 2015; Liu et al., 2018). However, the light energy of dwarf legume canopy is limited compared with sole cropping because of the shelter by higher crop canopy (Huang et al., 2022), which leads to restricted photosynthesis and low yield (Keating and Carberry, 1993; Feng et al., 2019). The different row ratio settings and strip widths of dwarf crops in intercropping have important effects on the microclimate environment, crop yield, and economic benefits in cereal and legume intercropping systems (Liu et al., 2018; van Oort et al., 2020; Wang et al., 2021a). Scientific and reasonable ratios can improve light energy interception and utilization efficiency, give full play to the advantages of high-position crops, and stimulate low-light response mechanisms in dwarf crops, thus promoting the yield improvement of intercropping systems to the greatest extent.

Intercropping results in a more complex canopy structure. The distribution and quality of light in the microclimate environment of crops canopy are crucial to crop photosynthesis and yield (Raza et al., 2019). In the intercropping compound system, the population light distribution and light transmittance have significant differences, which increase the light transmittance of high-position crops and reduce the light transmittance of low-position crops. The studies indicated that the intercropping improved the chlorophyll content and delayed the senescence process of high-position crops and promoted the net photosynthetic efficiency of border rows and nutrition utilization during the symbiotic period (Nasar et al., 2022). However, the negative intercropping productivity caused by interspecific competition has attracted more attention (Wu et al., 2016), especially in inappropriately managed fields. The canopy light extinction coefficient (*k*) of peanut was significantly decreased when intercropped with maize, while the mean radiation-use efficiency (*ε*) was significantly higher compared to sole peanut (Awal et al., 2006). Meanwhile, compared with monoculture, the yield of maize was increased by 61.05% in the maize–peanut intercropping system, whereas the yield of intercropped peanut was decreased by 31.80% (Li et al., 2019). Similarly, in the maize–soybean relay intercropping system, the leaves of soybean showed lower leaf mass per unit area, thinner thickness, lower chlorophyll a/b ratio, and lower photosynthetic rate during shade period (Wu et al., 2016). The application of wide strips for dwarf crops in intercropping systems was promoted to improve canopy light radiation and to be suitable for simplified planting (Brooker et al., 2015; van Oort et al., 2020). The light interception (LI) and light use efficiency (LUE) of intercropping peanut strip are significantly affected by the ratio of side rows in the maize and peanut strip intercropping system, and the relative yield of peanut is improved with the strip being wider (Wang et al., 2020). Therefore, it is one of the important ways to obtain yield advantage to improve the light environment through intercropping and row ratio allocation to achieve multi-level and all-around efficient utilization of light resources by the population (Wang et al., 2021a), and improve the efficiency of light energy utilization. Previous research has revealed the yield benefits of wide strips, but the mechanism underlying this improvement in photosynthetic characteristics of dwarf crops has not been well understood (Du et al., 2018).

Under the intercropping mode, there are significant differences in the photosynthetic effective radiation intensity and chlorophyll content of dwarf crop canopy (Kume et al., 2018; Wang et al., 2021b). The low-light environment of intercropping has become an important factor that inhibits the growth, development, and yield improvement of dwarf crops (Liu et al., 2017), because of the decrease of chlorophyll content per unit area and photosynthesis capacity in dwarf crops (Gong et al., 2020). In particular, the ratio of red light to far-red light in intercropping soybean canopy is significantly lower than that of monocropping, which caused soybean shading reaction yield reduction compared with monocropping (Yang et al., 2014). As the main pigment in plant photosynthesis, chlorophyll synthesis is not only regulated by internal genes, but also influenced by external environment. Insufficient and excessive light will inhibit chlorophyll synthesis, resulting in changes in chlorophyll content and composition. Owing to the reflected and absorbed effect by maize plants, the spectral irradiance, R/FR ratio, and photosynthetically active radiation (PAR), δ_Ro_ (the efficiency/probability with which an electron from the intersystem electron carriers was transferred to reduce end electron acceptors at the PSI acceptor side), and φ_Ro_ (the quantum yield for the reduction of the end electron acceptors at the PSI acceptor side) of intercropped soybean leaf were decreased compared with monocrop soybean, which resulted in the lower photosynthetic capacity in the maize–soybean intercropping system (Yao et al., 2017). It was also reported that, although the chlorophyll content and Chla/b of intercropped peanut decreased significantly, more Chla transformed into Chlb, which was conducive to absorbing short wave light, capturing more light energy, and improving the accumulation of photosynthetic products (Gong et al., 2015). The disadvantage of light competition significantly increased the rate of peanut falling and reduced the number of pods per plant and pods yield (Block et al., 2002; Feng et al., 2020). The limitation of photosynthetic synthesis and distribution is the limiting factor for the further improvement of the yield of the maize–peanut intercropping system (Jiao et al., 2021). Therefore, it is necessary to understand the importance of spatial and temporal allocation to improve the production of intercropped peanut and the advantages of intercropping systems (Gao et al., 2022).

In this study, 2 years of field experiments were conducted to explore the effects of different row ratios on the photosynthetic effective radiation, photosynthetic physiological characteristics, chlorophyll content, dry matter accumulation and distribution, yield, and its components of peanut canopy. The purpose is to compare the differences in light environment characteristics of peanut canopy in maize–peanut intercropping systems with different row ratios. The effects of interspecific competition on dry matter accumulation and yield of peanut were analyzed by measuring the photosynthetic physiological characteristics and chlorophyll synthesis law of intercropped peanut. According to the changes of photosynthetic physiological characteristics of peanut and the formation of yield advantage, a theoretical basis was provided for exploring the optimal maize interplanting model.

## 2 Materials and methods

### 2.1 Site description

**Figure 1:**
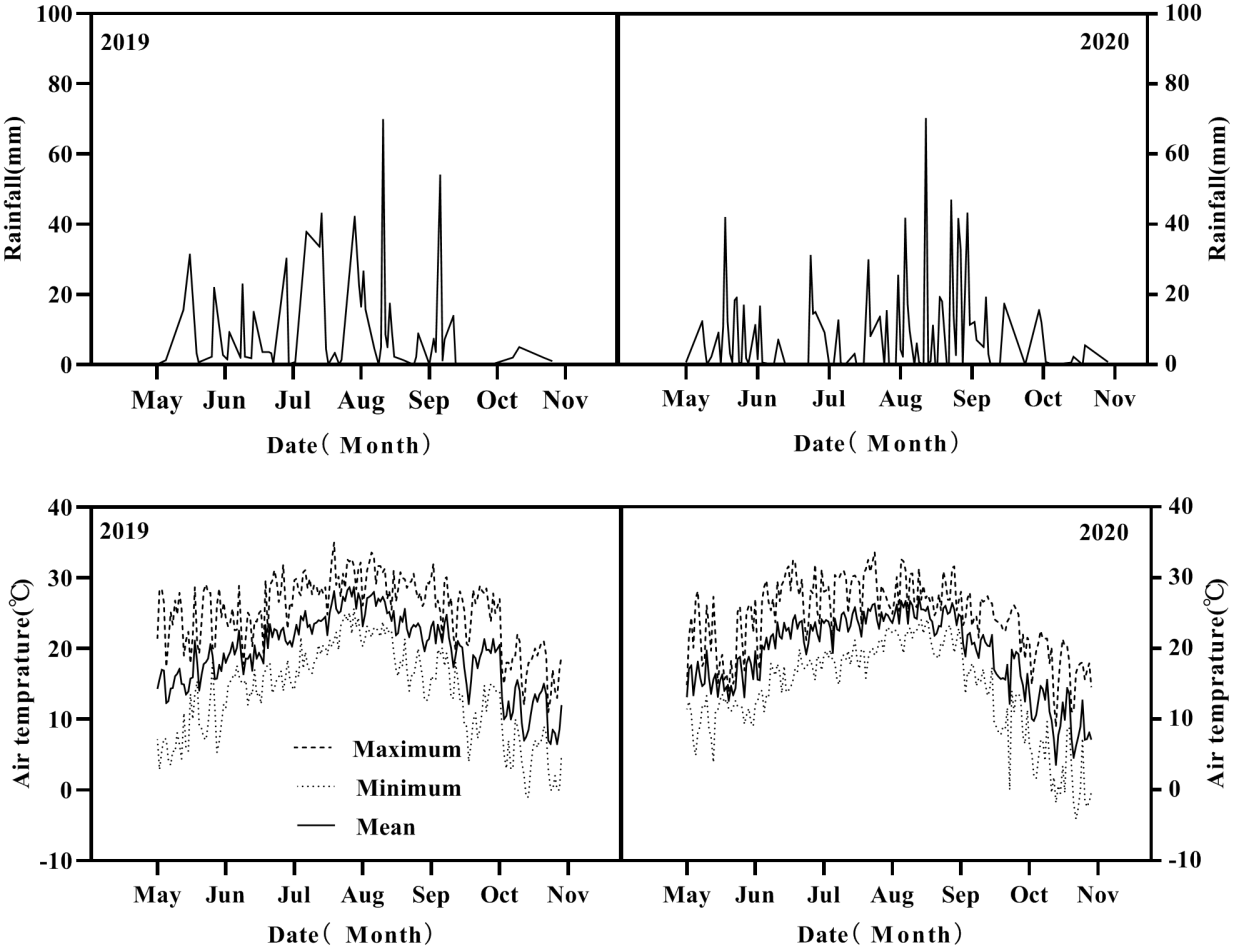
The air temperature and rainfall in the growing season of intercropping in 2019 and 2020.

Two-year experiments were conducted in the test field of Northeast Experimental Shenyang Agricultural Observation Station, Ministry of Agriculture and Rural Affairs (40°28′16″N, 124°06′45″E), Dandong city, Liaoning province, China during the 2019 and 2020 growing seasons (May to September). The previous crop was maize, and the soil physicochemical properties were as follows: soil organic matter, 18.4 g kg^−1^; available phosphorus, 42.2 mg kg^−1^ measured by the Olsen-p method; available potassium, 122.1 mg kg^−1^; alkaline hydrolyzable nitrogen, 86.0 mg kg^−1^; and soil pH, 6.4. The field location has a temperate monsoon continental climate, the average annual precipitation was 876.5 mm, and temperature was 10.8°C during the growth stage (**Figure 1**). Climate data were obtained from the Dandong Meteorological Bureau.

### 2.2 Experimental design

**Figure 2:**
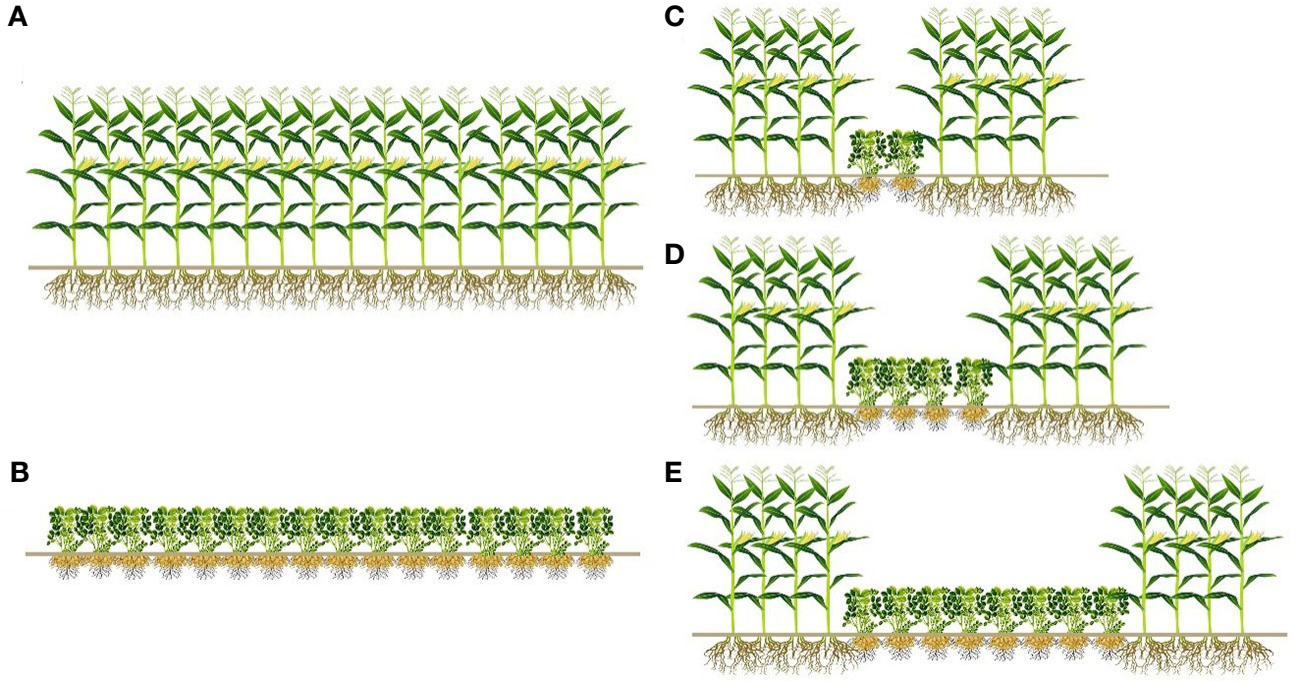
The layout of different row ratio configurations in intercropping of maize and peanut. **(A)** Sole maize (SM); **(B)** sole peanut (SP); **(C)** four rows of maize intercropping with two rows of peanut (M4P2); **(D)** four rows of maize intercropping with four rows of peanuts (M4P4); **(E)** four rows of maize intercropping with eight rows of peanuts (M4P8).

Field experiments were conducted using a randomized complete block design. This experiment included the following five treatments: sole maize (SM) and sole peanut (SP) consisting of 16 rows, and 4 rows of maize intercropped with 2 rows of peanuts (M4P2), with 4 rows of peanuts (M4P4), and with 8 rows of peanuts (M4P8), with three replicates per treatment, as shown in **Figure 2**. Maize variety was Liangyu 99 selected by Dandong Denghai Seed Industry Co. Ltd., China, and the peanut variety was Nonghua11 selected by the Peanut Research Institute of Shenyang Agricultural University, China. The row distances of sole crop and intercropping was 0.55 m, and the length of the test plot was 50 m. The planting density of sole and intercropped maize was 7.5 × 10^4^ plants ha^−1^, and the planting density of sole and intercropped peanut was 1.5×10^5^ plants ha^−1^. Sowing was performed with direct seeding on 5 May 2019 and 7 May 2020. Harvesting was performed on 5 October 2019 and 6 October 2020. For intercropping and sole cropping, the amount of fertilizer applied to maize and peanut was the same. A compound fertilizer (contained 14% N, 16% P_2_O_5_, and 15% K_2_O) was used as a basal fertilizer at a dose of 450 kg ha^−1^ at sowing time. There was no other form of fertilizer input during the growth period.

### 2.3 Measurements

#### 2.3.1 Photosynthetically active radiation

According to the method recommend by Yang et al. (2014), the average PAR of the middle of the plot and adjacent to maize and peanut canopy (50 cm above the ground) was measured using a light meter (AccuPAR LP-80, United States) between 9:00 and 16:00 h on a clear sunny day. Measurements were taken at the anthesis stage, podding stage, and maturity stage of peanut, repeated three times.

#### 2.3.2 Photosynthetic parameters

According to the method recommend by Wang et al. (2017) at the anthesis stage, podding stage, and maturity stage, photosynthetic parameters (*Pn*, *Ci*, *Tr*, and *Gs*) of the top three leaves on the main stem of peanut were measured at intervals of 2 h from 08:30 to 16:30 h with the LI-6400 XT portable photosynthesis system (LI-COR Inc., Lincoln, USA) equipped with a 2 cm × 3 cm clear chamber on a clear sunny day. The temperature and CO_2_ concentration of the leaf chamber resembled the natural environment. The sampling location was in the middle of the plot and adjacent to maize, repeated three times.

#### 2.3.3 Photosynthetic response curve

The photosynthetic response curves of the top three leaves of the peanut main stem were measured using the LI-6400 XT portable photosynthesis system (LI-COR Inc., Lincoln, USA). The parameters were measured on functional leaves from 09:00 to 11:30 h on a clear sunny day. The temperature and CO_2_ concentration of leaf chamber were maintained at 25°C and 380 µmol mol^−1^, respectively. PAR was increased from 0 to 1,500 µmol photons m^−2^ s^−1^ (0, 20, 50, 80, 100, 200, 400, 600, 800, 1,000, 1,200, and 1,500 µmol m^−2^ s^−1^, 36 min). The sampling location was in the middle of the plot and adjacent to maize, repeated three times.

#### 2.3.4 Chlorophyll content

Chla and Chlb contents were determined using the method of Guo et al. (2018) with slight modifications. Peanut leaves (0.2 g) were added to 80% acetone solution, shaken well, and extracted in the dark for 12 h. The optical density (OD) values of the extracts were measured at 663 nm and 645 nm using 80% acetone solution as a control to calculate the chlorophyll a, chlorophyll b, chlorophyll (a+b) content, and the chlorophyll a/b values. The sampling was the upper three leaves of the main stem of peanut and was collected at the anthesis stage, podding stage, and maturity stage, respectively, located in the middle of the plot adjacent to the maize, and repeated three times.

#### 2.3.5 Chlorophyll precursor content

δ-aminolevulinic acid (ALA) content was determined according to the method of Kumar Tewari and Charan Tripathy (1998) and Dalal and Tripathy (2012), with a slight modification: Fresh leaves (2 g) were ground with 6 ml of sodium acetate buffer (pH 4.6) in an ice bath, boiled in water for 15 min, centrifuged at 10,000 *g* for 20 min, and washed two times with 4 ml of extract. Supernatant (1 ml) was extracted, four drops of acetyl ethyl acetate was added to a boiled water bath for 15 min, an equal volume of Izod reagent was added, the absorbance value (A) at 553 nm was measured after 15 min, ALA-HCl (Sigma) was used as the standard sample to make a standard curve, and the ALA content (nmol g^−1^ FW) was calculated. The sampling was the upper three leaves of the main stem of the peanut and was collected at the anthesis stage, podding stage, and maturity stage, respectively, located in the middle of the plot adjacent to the maize, and repeated three times.

ProtoIX, Mg-ProtoIX, and Pchl content were determined according to the method of Hodgins and Van Huystee (1986) with slight modifications: Take fresh leaves (0.3 g) and place them in a precooled mortar. Add 5 ml of 80% alkaline acetone (acetone: 0.1 mol/L ammonia = 8:1, V/V) and grind in an ice bath. Homogenize at 18,000×*g*, centrifuge at 4°C for 15 min, extract the supernatant, and dilute to 25 ml with 80% alkaline acetone. Then, determine the absorbance values A628, A590 and A575 at 628-nm, 590-nm, and 575-nm wavelengths using a spectrophotometer. Finally, calculate the concentration of each substance according to the following formula and calculate the content in the sample (μ mol g^−1^ FW).


ProtoIX=0.18016×A575−0.04036×A628−0.04515×A590

Mg−ProtoIX=0.06077×A590−0.01937×A575−0.003423×A628

Pchl=0.03563×A628+0.007225×A590−0.02955×A575

#### 2.3.6 Dry matter

Five representative peanut plants were selected in each treatment at the anthesis stage, podding stage, and maturity stage. The plant samples were divided into roots, stems, leaves, and pods. Then, samples were baked in an oven for 30 min at 105°C, dried to constant weight at 85°C, and weighed. For determination of the dry matter accumulation amount, the dry weight of each sample was measured with an electronic balance (Heeyii JE-301, Hangzhou, China). The dry matter distribution rate (DDR) was calculated using the following formulas described by:


$$DDR(\%)=\;\frac{DW}{TDW}\times 100\%$$

where DW is the dry weight of each organ and TDW is the total dry weight of each plant.

#### 2.3.7 Yield and LER

The length of the ridge was 3 m and all middle rows of the intercropped maize and peanut were harvested, whereas the plants of 3 m × 8 rows in the middle of SP and SM were harvested to calculate yields at the maturity stage in 2019–2020. Then, 10 representative peanut plants were selected to measure the number of pods per plant, the number of full fruits per plant, the weight of 100 fruits, the rate of kernels, and the weight of 100 kernels.


$$\text{LER}=\left({\text{Y}}_{\text{im}}/{\text{Y}}_{\text{mm}}\right)+\left({\text{Y}}_{\text{ip}}/{\text{Y}}_{\text{mp}}\right)$$

Y_im_ and Y_mm_ are the yields of the intercropped and sole maize, respectively, and Y_ip_ and Y_mp_ are the yields of the intercropped and sole peanut, respectively. LER > 1 denotes intercropping gain, and LER< 1 indicates intercropping loss.

#### 2.3.8 Data analysis

Analysis of variance (ANOVA) was used to assess treatment effects on yield, dry matter and photosynthetic parameters, and year effect on fitted parameters using SPSS 20 (IBM, USA). Least significant differences (LSDs) were used to separate treatment differences in means at the 0.05 level. The graphs were made using Sigma plot (Version 12, Systat Software).

## 3 Results

### 3.1 Changes in photosynthetically active radiation in intercropped peanut canopy

There were significant differences in PAR in the canopy of different peanut row ratios (**Table 1**). Intercropping decreased the PAR of peanut canopy, and the smaller the peanut row ratio, the greater the PAR reduction. Compared with SP, the M4P8, M4P4, and M4P2 treatments decreased the PAR (mean of 2 years) by 7.34%, 26.28%, and 35.78% at the anthesis stage, 7.32%, 22.71%, and 31.45% at the podding stage, and 7.14%, 21.74%, and 33.71% at the maturity stage, respectively. Compared with M4P4 and M4P2, the PAR of M4P8 has significant advantages, which increased respectively by 25.72% and 38.84%, 20.03% and 35.47%, and 18.69% and 40.08%.

**Table 1:** Effects of different peanut row ratio configurations on PAR of peanut canopy in intercropping of maize and peanut.

Year	Treatment	Anthesis	Podding	Maturity
**2019**	**SP**	1,296.03 ± 35.51a	1,372.77 ± 37.29a	1,482.69 ± 25.28a
	**M4P8**	1,210.56 ± 36.54b	1,269.44 ± 18.00b	1,395.96 ± 38.36b
	**M4P4**	976.31 ± 10.00c	1,058.92 ± 33.54c	1,159.94 ± 18.44c
	**M4P2**	871.64 ± 19.46d	968.13 ± 21.10d	991.70 ± 17.74d
**2020**	**SP**	1,219.97 ± 24.71a	1,339.43 ± 28.43a	1,457.95 ± 37.01a
	**M4P8**	1,121.36 ± 30.03b	1,246.80 ± 43.54b	1,335.04 ± 44.13b
	**M4P4**	879.82 ± 22.80c	1,037.45 ± 22.82c	1,140.83 ± 18.22c
	**M4P2**	746.63 ± 17.80d	891.81 ± 19.45d	957.70 ± 36.21d
***p***	**Treatment**	***	***	***
	**Year**	ns	ns	ns
	**Treatment × Year**	ns	ns	ns

Data are expressed as the mean of three replicates ± standard error (n = 3), and different letters indicate statistical difference significance at p< 0.05 among the treatments by LSD tests. *** significant at 0.001 level, ns is not significant. SP: Sole peanut; M4P2: four rows of maize intercropped with two rows of peanut; M4P4: four rows of maize intercropped with four rows of peanuts; M4P8: four rows of maize intercropped with eight rows of peanuts.

### 3.2 Changes in chlorophyll content and composition in intercropped peanuts

**Figure 3:**
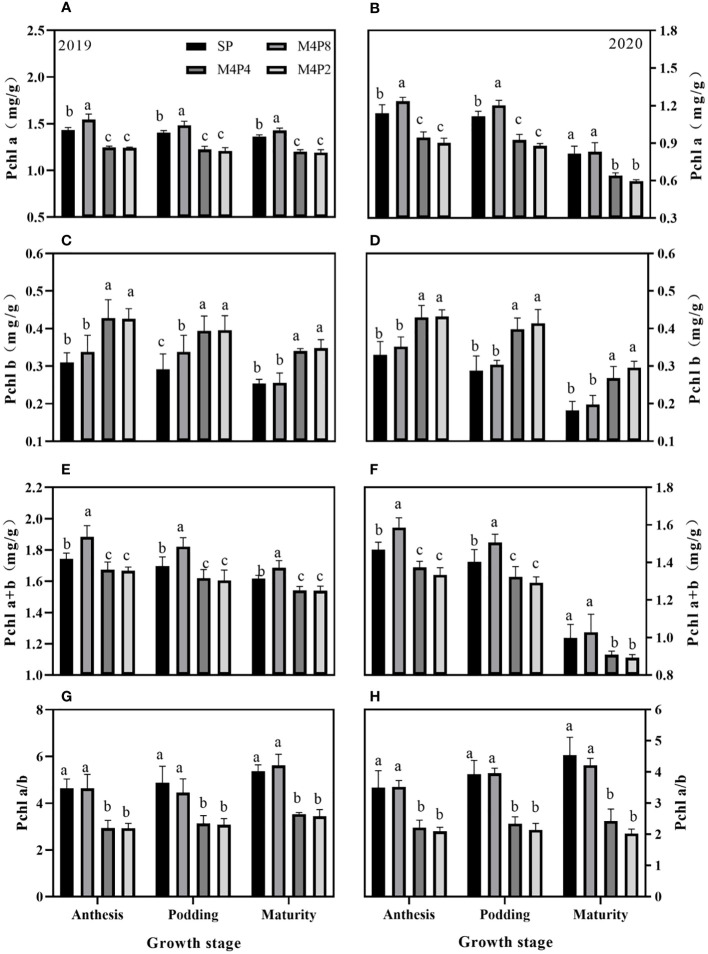
Effects of different peanut row ratio configurations on chlorophyll content in intercropped peanut. Different letters indicate statistical difference significance at *p*< 0.05 among the treatments by LSD tests. SP: Sole peanut; M4P2: four rows of maize intercropped with two rows of peanut; M4P4: four rows of maize intercropped with four rows of peanuts; M4P8: four rows of maize intercropped with eight rows of peanuts.

The peanut row ratio configurations had different effects on the chlorophyll content of peanut leaves (**Figure 3**). Chla and Chl(a+b) contents of M4P8 were significantly higher than SP, M4P4, and M4P2. Compared with the SP, M4P4, and M4P2 treatments, the M4P8 treatment increased the Chla content (mean of 2 years) by 8.20%, 27.40%, and 30.65% at the anthesis stage, 6.71%, 25.43%, and 29.77% at the podding stage, and 3.28%, 24.33%, and 29.62% at the mature stage, respectively. The Chl(a+b) content (mean of 2 years) increased by 8.10%, 20.26%, and 22.40% at the anthesis stage, 11.06%, 13.11%, and 15.01% at the podding stage, and 3.61%, 11.28%, and 12.37% at the mature stage, respectively. Compared with SP, the Chl(a/b) value (mean of 2 years) of M4P8 had no significant change in the three growth stages, but were 58.42%, 55.58%, and 66.38% and 62.77%, 65.05%, and 85.95% higher than that of M4P4 and M4P2, respectively. Intercropping increased the content of Chlb, but there was no significant difference between M4P8 and SP (except for 15.7% higher than SP at the anthesis stage in 2019), which was lower than that of M4P4 and M4P2.

### 3.3 Changes in chlorophyll precursors in intercropped peanuts

**Figure 4:**
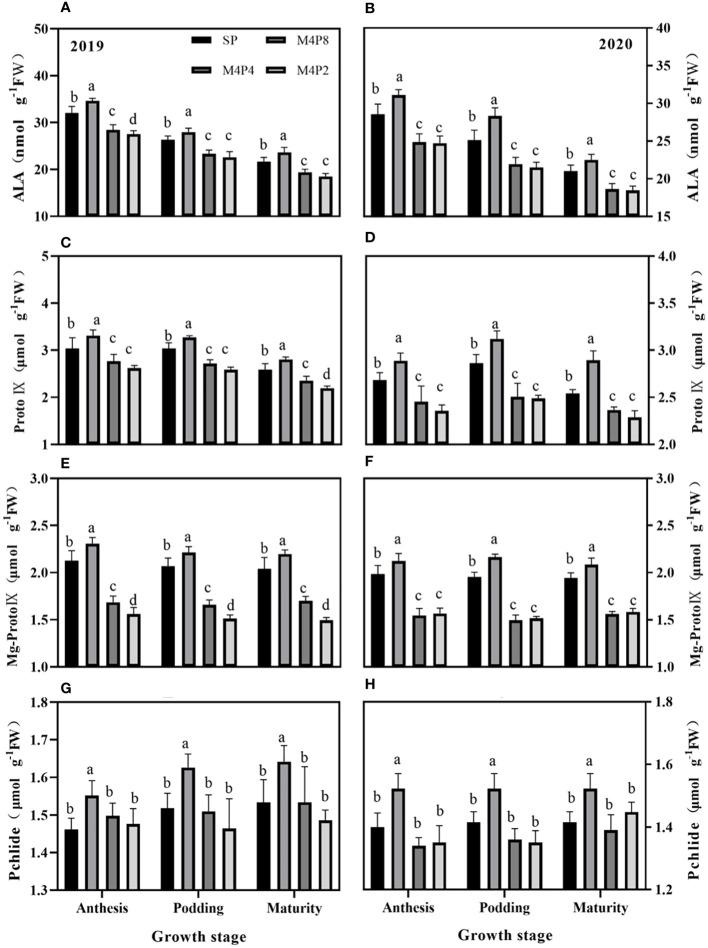
Effects of different peanut row ratio configurations on the content of chlorophyll synthesis precursors in intercropped peanut. Different letters indicate statistical difference significance at *p*< 0.05 among the treatments by LSD tests. SP: Sole peanut; M4P2: four rows of maize intercropped with two rows of peanut; M4P4: four rows of maize intercropped with four rows of peanuts; M4P8: four rows of maize intercropped with eight rows of peanuts.

Intercropping peanut row ratio configurations affected the synthesis of peanut chlorophyll precursors and their conversion to chlorophyll. Overall, ALA, Proto IX, Mg-Proto IX, and Pchlide were significantly higher in M4P8 treatment than in SP; however, it was significantly lower in M4P2 and M4P4 treatment (**Figure 4**). The ALA content (2-year mean) of M4P8 was 8.51%, 9.46%, and 7.99% higher than SP (**Figures 4A, B**); the Proto IX content was 8.35%, 8.43%, and 11.14% higher than SP (**Figures 4C, D**); the Mg-Pto IX content was 7.70%, 8.91%, and 7.52% higher than SP (**Figures 4E, F**); and the Pchlide content was 7.44%, 7.30%, and 6.87% (**Figures 4G, H**) higher than SP at the flowering needle stage, pod stage, and maturity stage, respectively.

### 3.4 Diurnal variation of photosynthetic characteristics in intercropped peanut

**Figure 5:**
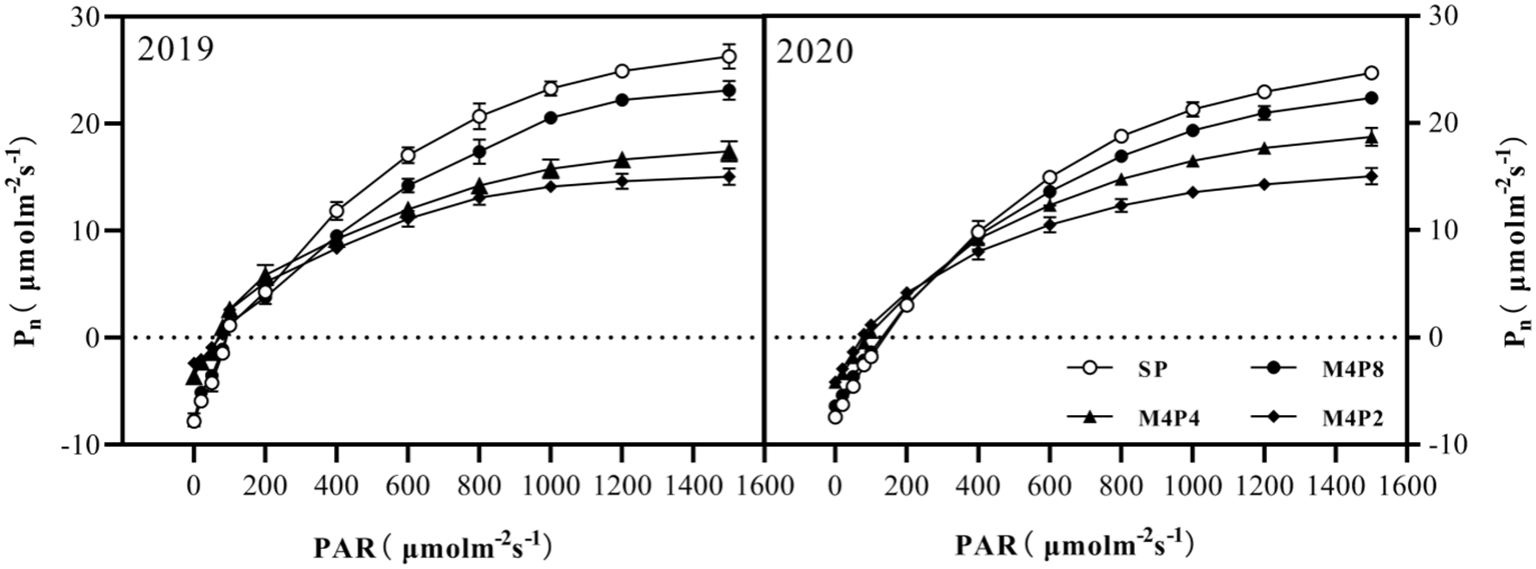
Effects of different peanut row ratio configurations on photosynthetic response curve of intercropped peanut. Different letters indicate statistical difference significance at *p*< 0.05 among the treatments by LSD tests. SP: Sole peanut; M4P2: four rows of maize intercropped with two rows of peanut; M4P4: four rows of maize intercropped with four rows of peanuts; M4P8: four rows of maize intercropped with eight rows of peanuts.

**Figure 6:**
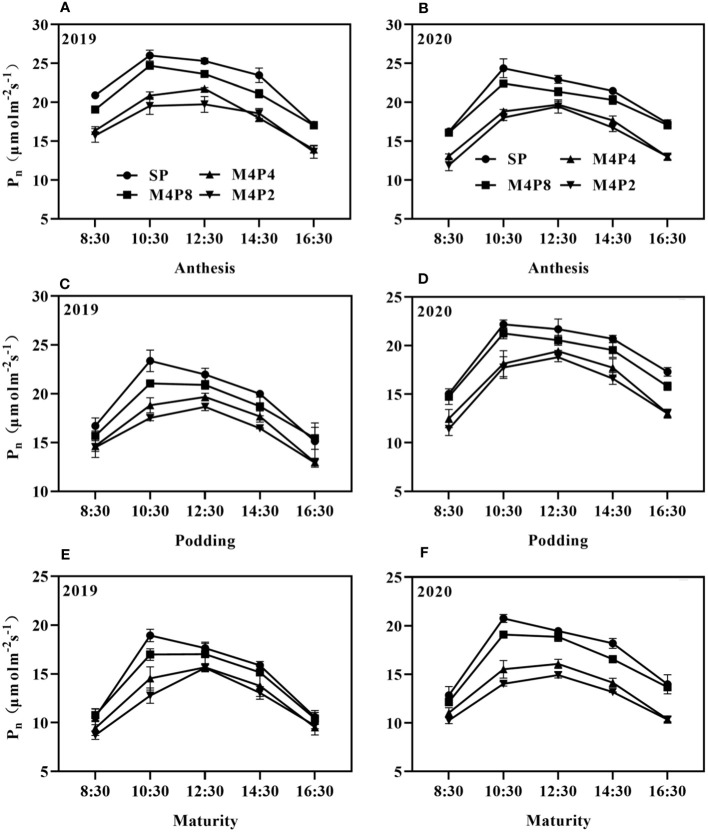
Effects of different peanut row ratio configurations on diurnal variation of net photosynthetic rate of intercropped peanut. Different letters indicate statistical difference significance at *p*< 0.05 among the treatments by LSD tests. SP: Sole peanut; M4P2: four rows of maize intercropped with two rows of peanut; M4P4: four rows of maize intercropped with four rows of peanuts; M4P8: four rows of maize intercropped with eight rows of peanuts.

**Figure 7:**
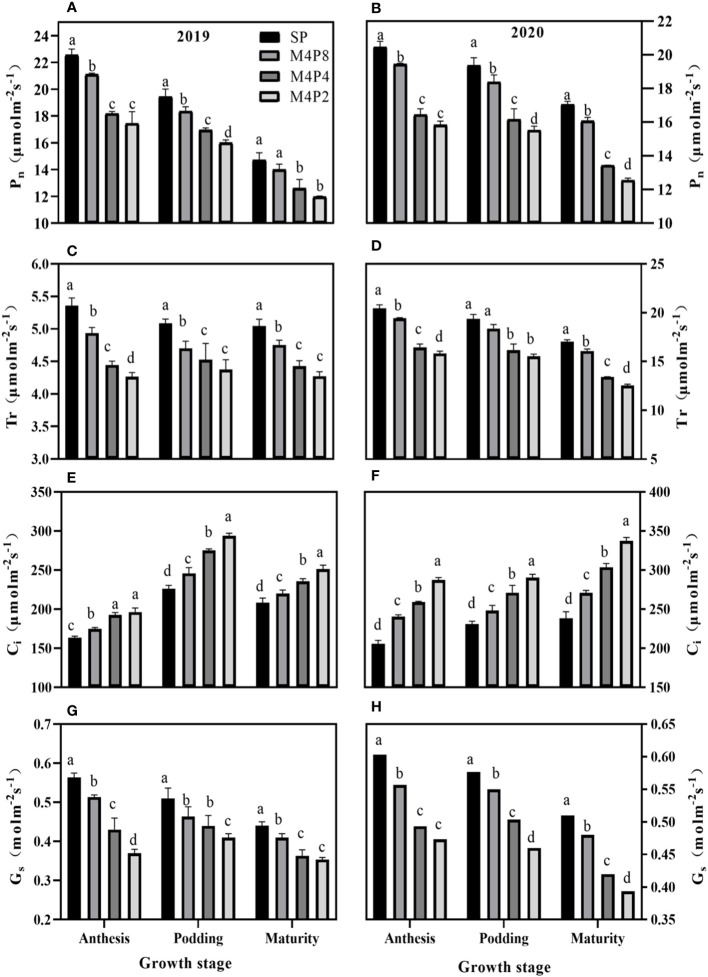
Effects of different peanut row ratio configurations on photosynthetic parameters of intercropped peanut. Different letters indicate statistical difference significance at *p*< 0.05 among the treatments by LSD tests. SP: Sole peanut; M4P2: four rows of maize intercropped with two rows of peanut; M4P4: four rows of maize intercropped with four rows of peanuts; M4P8: four rows of maize intercropped with eight rows of peanuts.

*Pn* in peanut leaves increased rapidly with the increase of light intensity. When PAR reached 600 mol m^−2^ s^−1^, the increase of *Pn* began to slow down and gradually approached the saturation state (**Figure 5**). At lower PAR, *Pn* was higher in intercropping peanuts; at higher PAR, *Pn* was higher in sole peanut (**Figure 6**). The diurnal variation of *Pn* all showed a single peak curve and was affected by the peanut row ratio configurations. Compared with SP, the M4P4, M4P2, and M4P8 treatment showed significantly lower maximum photosynthetic rates (2-year average), whereas the M4P8 treatment exhibited the smallest decrease and a relatively longer duration of high photosynthetic rates. Compared with SP, *Pn*, *Tr*, and *Gs* were significantly decreased in all three cropping patterns, with M4P8 showing the least reduction, followed by M4P4, and M4P2 with the greatest reduction (**Figures 7A, B, G, H**). Of these, the M4P8 pattern *Pn* (2-year mean) was reduced by 5.68%, 5.33%, and 5.30%; *Tr* by 7.41%, 5.45%, and 5.95%; and *Gs* by 8.20%, 6.88%, and 6.46% compared to SP, at the flowering needle stage, pod stage, and maturity, respectively. Conversely, intercropping promoted an increase in *Ci*, and this increased as the peanut row ratio decreased (**Figures 7E, F**), with the smallest increase in M4P8, followed by M4P4, and the largest in M4P2. Compared to SP, *Ci* increases by 11.95%, 8.06%, and 9.61% in M4P8 mode at the flowering needle stage, pod stage, and maturity, respectively.

### 3.5 Changes in dry matter accumulation and distribution rates of intercropped peanuts

**Figure 8:**
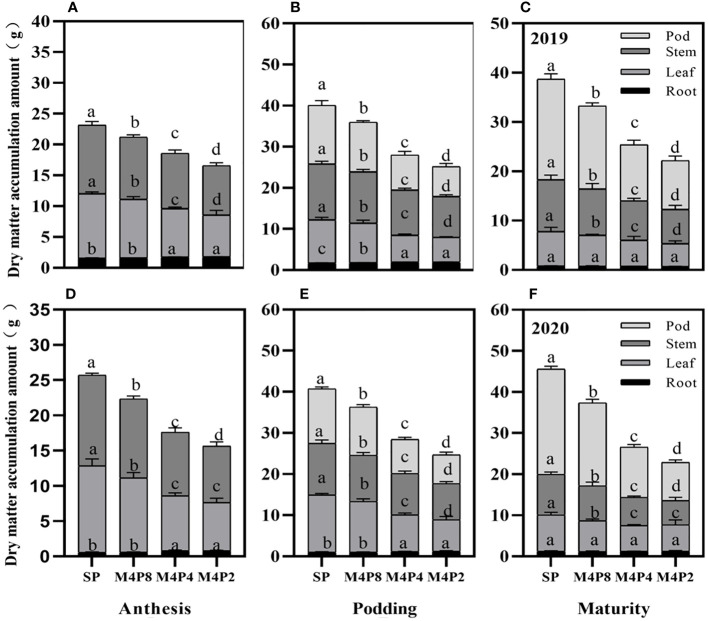
Effects of different peanut row ratio configurations on dry matter accumulation in intercropped peanut. Different letters indicate statistical difference significance at *p*< 0.05 among the treatments by LSD tests. SP: Sole peanut; M4P2: four rows of maize intercropped with two rows of peanut; M4P4: four rows of maize intercropped with four rows of peanuts; M4P8: four rows of maize intercropped with eight rows of peanuts.

The accumulations of per-plant and organ dry matter were significantly varied among the different treatments (**Figure 8**). The dry matter per plant in each growth stage was SP > M4P8 > M4P4 > M4P2, which reached a significant difference among different treatments. Compared with SP, the dry weight per plant of M4P8 (2-year mean) was decreased by 10.77%, 10.55%, and 15.92% at the anthesis stage, podding stage, and mature stage, respectively.

Intercropping significantly reduced the dry matter of pods, stems, and leaves at each stage, but there was no significant difference in root dry weight between M4P8 and SP treatments (**Figure 8**). All organs of M4P8 treatment were significantly higher than those of M4P4 and M4P2 treatments (except root dry weight at mature stage). The increase of intercropped peanut rows was beneficial to the accumulation of dry matter per plant and each organ.

**Figure 9:**
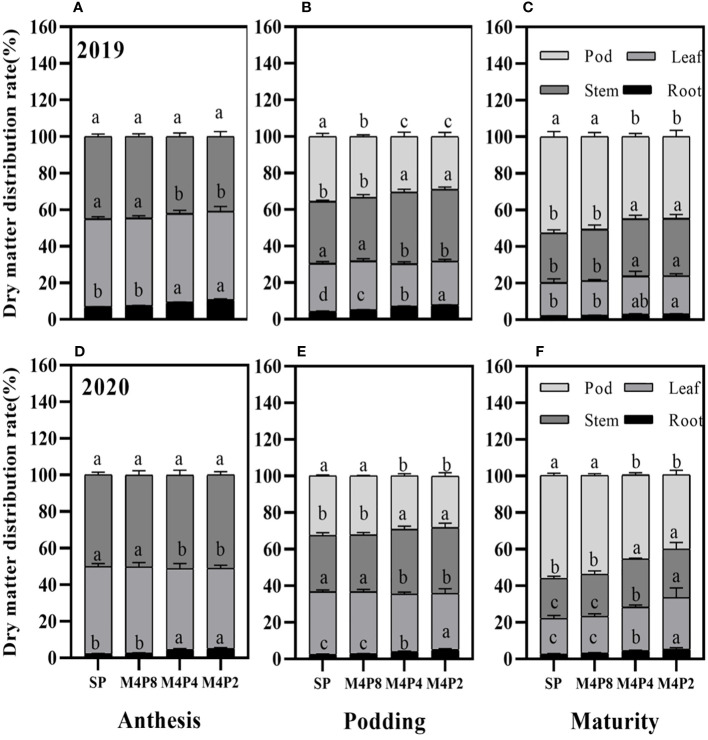
Effects of different peanut row ratio configurations on dry matter distribution of intercropped peanut. Different letters indicate statistical difference significance at *p*< 0.05 among the treatments by LSD tests. SP: Sole peanut; M4P2: four rows of maize intercropped with two rows of peanut; M4P4: four rows of maize intercropped with four rows of peanuts; M4P8: four rows of maize intercropped with eight rows of peanuts.

The dry matter distribution ratios of organs were significantly different at each growth stage (**Figure 9**), which was affected by the intercropping peanut row ratio. Compared with SP, there were no significant differences in dry matter distribution ratios among organs in M4P8 treatments at the anthesis stage, podding stage, and mature stage, respectively (**Figure 9**). However, compared with SP, there were significant differences in dry matter distribution ratios among organs in M4P4 and M4P2. In particular, the dry matter distribution of M4P4 and M4P2 peanut pods was reduced by 12.24% and 15.59% and 16.47% and 21.23% at podding and harvest, respectively.

### 3.6 Changes in yield and compositional of intercropping peanuts

Compared with SP, the yields of intercropped peanut were decreased, while the land use efficiency was significantly increased because the LERs of the three intercropping treatments were more than 1. The highest in M4P8 were 1.34 in 2019 and 1.31 in 2020, which were significantly higher than M4P4 and M4P2 (**Table 2**). With the increase of peanut row ratio, the pod yield of peanut was increased. Compared with SP, the yield of M4P8 treatment was decreased by 36.65%, but it is increased by 40.99% and 79.01%, respectively, compared with the M4P4 and M4P2 treatments. Compared with SP, the number of pods per plant in the intercropped peanut of M4P8, M4P4, and M4P2 treatments were decreased by 7.56%, 20.11%, and 26.08%. Compared with SP, the number of full pods in the M4P8, M4P4 and M4P2 treatments were decreased by 11.18%, 23.72%, and 30.45%, respectively. Compared with SP, the 100-kernel weight in the M4P8, M4P4, and M4P2 treatments were decreased by 5.93%, 13.44%, and 19.45%, respectively. Compared with SP, 100-seed weight in the M4P8, M4P4, and M4P2 treatments was decreased by 5.84%, 13.14%, and 16.98%, respectively. Compared with SP, the kernel ratio in the M4P8, M4P4, and M4P2 treatments was decreased by 8.98%, 16.99%, and 22.64%, respectively.

**Table 2:** Effects of different peanut row ratio configurations on yield and yield components and LER in intercropping of maize and peanut.

	Treatment	Yield components					Yield (kg ha^−1^)		LER
		Pod number	Full Pod number	100-kernel weight (g)	100-seed weight (g)	Kernel ratio (%)	Peanut	Maize	
**2019**	SP	24.45 ± 0.59a	14.89 ± 0.67a	168.82 ± 4.77a	65.38 ± 2.36a	48.7 ± 0.22a	4,144.73 ± 117.99a	11,077.68 ± 82.85a	1.00
	M4P8	23.10 ± 0.55a	13.11 ± 0.51b	158.71 ± 3.71a	60.68 ± 2.32a	44.6 ± 0.32b	2,634.15 ± 137.82b	7,843.63 ± 563.65b	1.34 ± 0.01a
	M4P4	20.23 ± 0.77b	11.44 ± 0.59c	145.83 ± 3.85b	54.49 ± 1.46b	40.3 ± 0.42c	1,924.42 ± 112.17c	8,399.73 ± 457.21b	1.22 ± 0.01b
	M4P2	19.11 ± 0.67b	10.89 ± 0.45c	137.11 ± 5.58b	53.41 ± 1.81b	38.3 ± 0.12d	1,485.67 ± 82.31d	10,258.87 ± 458.88a	1.25 ± 0.01b
	Mean	21.72	12.58	152.62*	58.49	42.98	2,547.24*	9296.58	1.27
**2020**	SP	26.78 ± 0.64a	16.00 ± 0.59a	161.34 ± 3.18a	62.62 ± 1.58a	55.6 ± 0.23a	3,771.28 ± 74.65a	13,365.16 ± 560.96a	
	M4P8	25.67 ± 0.50a	14.33 ± 0.35b	151.86 ± 3.16b	59.81 ± 1.47ab	50.3 ± 0.63b	2,381.29 ± 115.76b	9,007.53 ± 423.09b	1.31 ± 0.01a
	M4P4	22.00 ± 0.50b	12.11 ± 0.48c	139.94 ± 2.17c	56.60 ± 2.66bc	46.3 ± 0.52c	1,641.07 ± 96.22c	9,966.32 ± 616.48b	1.18 ± 0.01b
	M4P2	19.89 ± 0.37c	10.56 ± 0.40d	128.88 ± 1.33d	52.82 ± 2.10c	42.3 ± 0.53d	1,317.64 ± 109.29b	11,187.07 ± 458.54a	1.19 ± 0.01b
	Mean	23.58*	13.25	145.51	57.96	48.63*	2,277.82	10881.52*	1.22
**Means (2-year average)**	SP	25.62 ± 0.66a	15.44 ± 0.44a	165.08 ± 2.87a	64.00 ± 1.35a	52.15 ± 0.98a	3,958.00 ± 90.30a	12,221.42 ± 474.36a	1.00
	M4P8	24.38 ± 0.46a	13.72 ± 0.33b	155.29 ± 2.33b	60.24 ± 1.23a	47.45 ± 1.04b	2,507.72 ± 91.50b	8,425.58 ± 374.07b	1.32 ± 0.01a
	M4P4	21.12 ± 0.48b	11.78 ± 0.37c	142.89 ± 2.22c	55.54 ± 1.40b	43.3 ± 0.56c	1,782.74 ± 82.42c	9,183.03 ± 438.65b	1.20 ± 0.01b
	M4P2	19.50 ± 0.37c	10.72 ± 0.29d	133.00 ± 2.93d	53.12 ± 1.24b	40.3 ± 0.44d	1,401.66 ± 67.59d	10,526.17 ± 351.68a	1.22 ± 0.01b
	Treatment	***	***	***	***	***	***	***	***
***p***	Year	***	ns	*	ns	***	*	*	ns
	Treatment × Year	*	ns	ns	ns	ns	ns	ns	ns

Data are expressed as the mean of three replicates ± standard error (n = 3). and different letters indicate statistical difference significance at p< 0.05 among the treatments by LSD multiple range tests. * significant at 0.05 level, *** significant at 0.001 level, ns is not significant. SP: Sole peanut; M4P2: four rows of maize intercropped with two rows of peanut; M4P4: four rows of maize intercropped with four rows of peanuts; M4P8: four rows of maize intercropped with eight rows of peanuts.

Compared with 2020, the average values of pod number, kernel ratio, and yield of maize in 2019 were significantly higher, while the 100-kernel weight and yield of peak were significantly lower than those in 2020. There was no significant difference in other indicators. Compared with SP, the full pod number, 100-kernel weight, kernel ratio, and yield of peanut in M4P8 (mean of 2 years) were significantly reduced, while there was no significant difference in the 2-year average values of pod number and 100-seed weight. SP and M4P8 were significantly higher than M4P4 and M4P2.

## 4 Discussion

### 4.1 Intercropping changes the light distribution and photosynthetic physiological characteristics of peanut canopy

Light is the most important environmental factor among many external factors that influence the synthesis and accumulation of photosynthetic products of crops (Huang et al., 2022). PAR is the energy source of crop life activities, organic matter synthesis, and yield formation. Whether PAR is too high or too low, the photosynthetic capacity of plants will be reduced (Abakumova et al., 2016; Gou et al., 2017). The synthesis and distribution of peanut photosynthetic products were directly inhibited by the intercepted light, which is mostly side light, leading to significant changes in the light environment. Previous research reports found that different row ratio configurations (Wang et al., 2021a), row spacing (Zhang et al., 2015), and planting density (Mao et al., 2014; Yang et al., 2021) influenced the light transmittance of intercropping composite populations. Our study found that intercropping reduces the photosynthetic effective radiation reaching the peanut canopy to varying degrees, compared with monoculture. This is due to the fact that maize at a high position would produce a shading effect on peanuts, resulting in significant differences in population light distribution and light transmittance between different planting patterns. In this study, the degree of reduction in PAR in the peanut canopy was strongly correlated with the setting of the peanut row ratio; i.e., there was a significant advantage in light radiation in the M4P8 treatment and the peanut canopy PAR was significantly higher than in the M4P4 and M4P2 models (**Table 1**). The main reason was that the intercropping system has three-dimensional optical characteristics, which can promote the utilization efficiency of high-position crops for strong light and low-height crops for weak light, and realize the efficient utilization of light in the system (Wang et al., 2021a). This is consistent with previous studies in strip intercropping systems with different row ratio configurations that have found that the ability of two species to compete for light varies with ecological niche when the light environment of the system is changed (Zhang et al., 2015; Umesh et al., 2023). In the M4P8 pattern, interspecific competition diminished and peanut had a greater advantage in light radiation. These results demonstrate that row-ratio configuration is one of the principal factors that regulate the photosynthetic product synthesis and distribution of intercropped peanut.

Photosynthesis determines the future of agricultural production (Fan et al., 2019). Photosynthetic physiological characteristics have always been an important part of many scholars’ research. For instance, Gong et al. (2020) found that the *Pn* values of the upper, middle, and lower layers of the millet canopy were significantly higher than SP by 8.8%–32.5%, 16.0%–46.3%, and 25.0%–114.4% (*p<* 0.05), respectively, under the millet/mung bean intercropping system. Wang et al. (2021a) found that the LI of maize was 23.4% higher than that of the control, and the LI of shaded peanut was 32.2% lower in the study of maize and peanut strip intercropping. LI of intercropped maize increased with the increase of BRP. The LI of peanut decreased with the decrease of BRP, but there was no significant difference between the M6P6 and M8P8 treatment. Our study found that the *Pn*, *Tr*, and *Gs* decreased in intercropped peanut, while *Ci* increased in intercropped peanut (**Figure 7**). The decline in *Pn*, *Tr*, and *Gs* in intercropped peanut was due to shade from intercropped maize, which inhibited photosynthesis in peanut. While *Pn*, *Tr*, and *Gs* in the M4P8 treatment was lower than in sole peanut, they were significantly higher than in the M4P4 and M4P2 treatments (**Figure 7**), indicating that the row ratio configuration of the intercropping system was beneficial in alleviating the effects of shade from intercropped maize on the reduction of photosynthetic rates in the canopy leaves of peanut. Compared with sole peanut, *Ci* increased in the functional leaves of intercropped peanut, of which the smallest increase was observed in the M4P8 treatment. These results indicated that the decrease in photosynthetic capacity of intercropped peanut was caused by non-stomatal limitation (Gong et al., 2015; Yao et al., 2017; Han et al., 2022) and that the row ratio configuration of the M4P8 treatment was favorable to the interception and uptake of PAR by peanut leaves in the maize peanut intercropping system, thus improving the photosynthetic characteristics of the leaves.

### 4.2 Intercropping changed synthesis of chlorophyll and its precursor in peanut leaves

Chlorophyll is the main carrier of plant photosynthesis, and Chla determines photosynthesis, while Chlb determines the breadth of the spectrum utilized (Brestic et al., 2015). Studies have shown that intercropping increases the relative chlorophyll content of intercropped oats, peanuts, and soybeans (Yao et al., 2017; Bernas et al., 2021; Shi et al., 2021). Our study showed that different row ratio configurations changed chlorophyll content and photosynthetic efficiency in peanut, with the M4P8 pattern increasing the content of Chla, Chlb, and Chla+b in leaves, decreasing the ratio of Chla/b, and enhancing the efficiency of strong and weak light utilization (**Figure 3**). Under the M4P4 and M4P2 models, the content and proportion of Chlb increased, which enhanced the use of low light, but the Chla and Chla+b content was significantly reduced as a result of the high level and duration of shade, which was detrimental to the use of strong light and reduced photosynthetic capacity. It is clear that the intensity of light has a direct effect on the synthesis, the content, and the distribution of chloroplasts, and that peanut maintains cellular energy balance by regulating the structure and function of its photosynthetic machinery to adapt to changes in the environment (Li et al., 2019).

Chlorophyll synthesis is a series of enzymatic catalytic processes, and insufficient or too much light can affect chlorophyll biosynthesis (Banas et al., 2011; Ashraf and Harris, 2013; Wang et al., 2017). In this study, the change in trend of ALA content and chlorophyll content was consistent (**Figures 4A, B**), and the difference of ALA content was the important reason for the difference of chlorophyll content in peanut leaves under different intercropping. In SP, excessive illumination causes light inhibition, and Heme accumulation inhibits the synthesis of ALA. In contrast, the M4P8 pattern has relatively little effect of shade from maize to peanut, avoiding strong light inhibition and weak light stress to promote ALA synthesis, providing strong conditions for chlorophyll synthesis in this pattern.

The transformation of Proto IX into Mg Proto IX is an important branch of the chlorophyll synthesis pathway, and Mg proto IX is a sign that Proto IX enters the chlorophyll synthesis pathway (Kopečná et al., 2015; Liu et al., 2018). In this study, the results indicated that the contents of Proto IX and Mg Proto IX in the M4P8 pattern were significantly higher than those in SP (**Figures 4C–F**), which was consistent with the change in trend of ALA and chlorophyll content, indicating that chlorophyll synthesis did not change under this pattern. With the reduction of peanut row ratio, the decrease of Mg Proto IX content in M4P4 and M4P2 models was significantly greater than that in M4P8, which may be related to the strong canopy and long shading time, limiting the expression of MgPEC synthase gene and inhibiting chlorophyll biosynthesis. Moreover, studies found that the wheat root acid, citric acid, and other plant iron carriers secreted by maize roots can increase iron absorption of peanuts in maize and peanut intercropping (Xiong et al., 2013; Guo et al., 2014). More Proto IX combines with Fe^2+^ to form Heme. There is an obvious competition between Mg proto IX and Heme in the metabolic process of tetrapyrrole in plants. The heme produced by the combination of Proto IX and Fe^2+^ can regulate feedback and inhibit the synthesis of ALA (Yang et al., 1995). Hence, the conversion efficiency of Proto IX to Mg Proto IX decreased, resulting in the insufficient yield of Mg Proto IX and the accumulation of heme.

Our study found that compared with SP, the content of Pchlide did not significantly change under M4P4 and M4P2 (**Figures 4G, H**), but the content of its transformation product Chla and the previous product Mg Proto IX was significantly reduced. In the process of chlorophyll synthesis, a step of synthesis is blocked, its precursor substances will accumulate, and the subsequent precursor substances will decrease (von Gromoff et al., 2008). Our results indicated that Pchlide was blocked in the conversion process of synthetic chlorophyll a resulting in the reduction of chlorophyll content in M4P4 and M4P2, while this phenomenon does not occur in M4P8. The above results show that maize interplanting can promote the effective use of light energy by changing the row ratio configuration, thereby improving the photosynthetic capacity of leaves and the accumulation of the photosynthetic product.

These findings demonstrate that the negative effects on chlorophyll synthesis in peanut due to high intensity and duration of maize shade can be effectively reduced by increasing the number of rows of peanut in a strip intercropping system. Meanwhile, we hypothesize that, in practice, in a strip intercropping system, patterns such as four rows of maize and eight rows of peanuts can promote the effective use of light energy in the peanut canopy, which is conducive to chlorophyll synthesis in intercropped peanut leaves, thus improving the photosynthetic capacity of leaves and photosynthetic product accumulation.

### 4.3 Dry matter accumulation, distribution, and yield formation of intercropping composite population

The transfer of dry matter from other organs to the pod at the late growth stage determined the peanut yield. This study found that the dry matter accumulation and distribution rate between organs of intercropped peanut were lower than those of monocrop peanut, and these results were like those of intercropped maize and soybean (Yang et al., 2014). Furthermore, intercropping reduces the photosynthetic characteristics of peanut canopy, thereby inhibiting the accumulation and distribution of photosynthetic products, and the dry weight distribution ratio of each organ in M4P8 was significantly higher than that in M4P4 and M4P2 (**Figures 8**, **9**). This was partly due to the fact that proper row ratio configuration improved the photosynthetic characteristics of dwarf crop peanut. These results showed that the M4P8 treatment could increase the accumulation of dry matter among organs, improve the distribution of dry matter among organs, and then promote the transport of dry matter from vegetative organs to grains.

**Figure 10:**
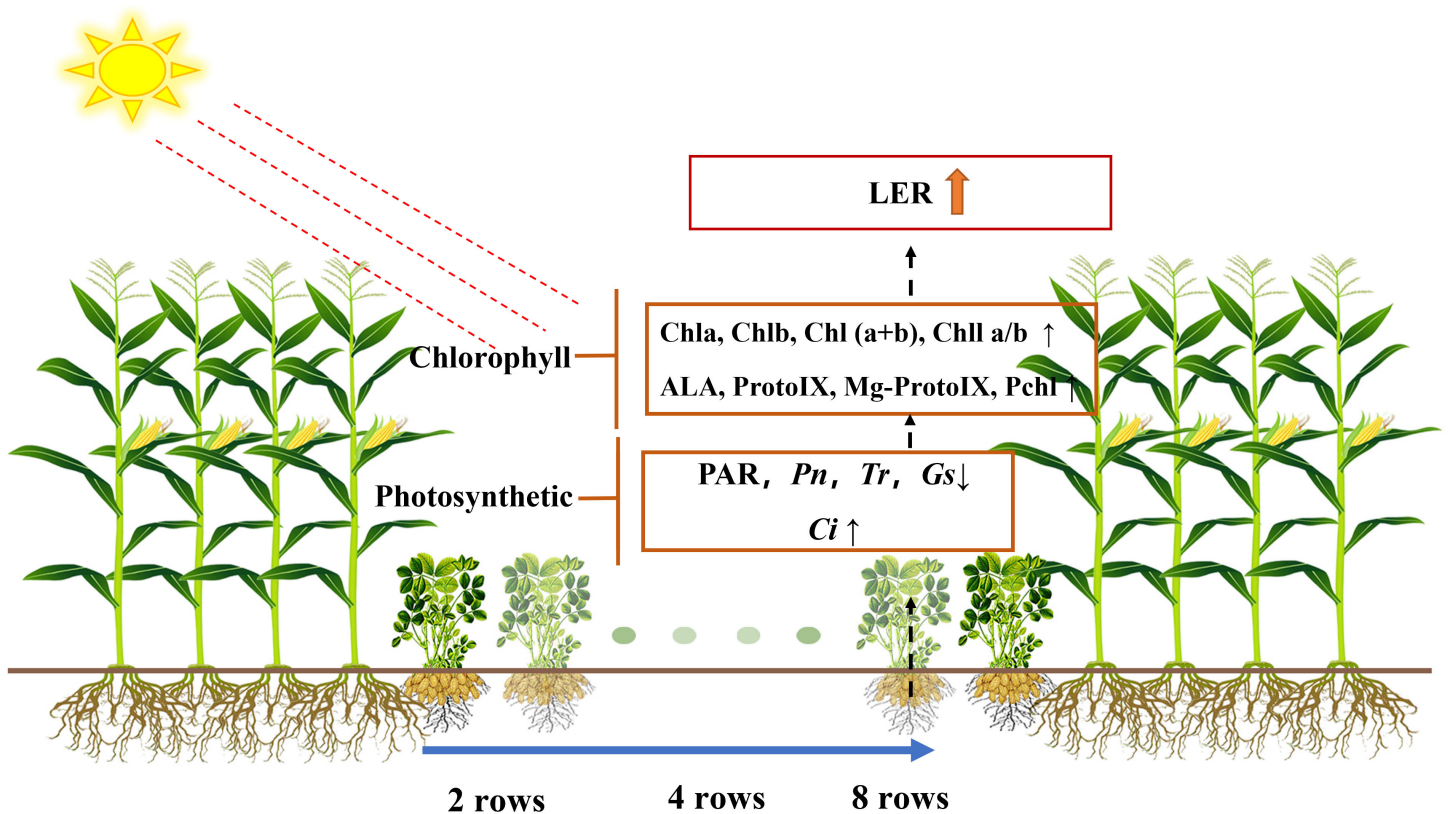
Effect of changes in photosynthetic characteristics of intercropped peanut with different row ratio configurations on intercrop yield and land equivalent ratio.

Intercropping had an overall yield advantage and improved the land use efficiency (Martin-Guay et al., 2018). In this study, under different intercropping treatments, LERs were greater than 1, indicating that the total yield of the intercropping system was increased within limited area (**Table 2**). Wangiyana et al. (2021) found more green leaves and greener leaves of intercropped sweetcorn compared to monocropped ones, which supported higher grain yield under intercropping (Wangiyana et al., 2021); this is consistent with our findings. The yield advantage of intercropping is mainly due to maize. Although the peanut pod relative yield in M4P8 was lower than that in monoculture, the yield and LER in M4P8 were the highest compared with M4P2 and M4P4 (**Figure 10**). These results indicated that increasing row ratio of intercropped peanut could optimize population structure, reduce shading effects by maize, and improve light energy utilization rate and dry matter accumulation of the population (Wang et al., 2020). It could be seen that the reasonable configuration alleviated the yield reduction caused by the inferior position of dwarf crops in the intercropping system (Zhang et al., 2015). Our study found that the full pod number decreases as the peanut row ratio decreases and increases with year, but there is no significant difference between years. In M4P8, the yield components were significantly better than other intercropping modes, and the optimization effect was relatively ideal. Therefore, it was necessary to appropriately increase the number of peanut planting lines in the maize peanut intercropping system, improve the interspecific competitiveness of peanut, and ensure the yield advantage of the intercropping system.

## 5 Conclusion

Different peanut row ratio settings change the peanut canopy PAR. The difference of PAR under different intercropping modes affects the photosynthetic physiological characteristics of peanut. The very small intercropping peanut row ratio hinders the synthesis of peanut chlorophyll. The insufficient synthesis of ALA, the reduction of the conversion efficiency of Proto IX to Mg-Proto IX, and the obstruction of Pchlide in the conversion process of synthesizing chlorophyll a are the root causes of the difference, thus reducing the photosynthetic capacity of peanut functional leaves, affecting the yield of the intercropping system. As the best row ratio configuration of maize intercropping, the M4P8 model has significant yield advantages, improves land use efficiency, and contributes to sustainable agriculture.

## Data availability statement

The original contributions presented in the study are included in the article/supplementary material. Further inquiries can be directed to the corresponding authors.

## Author contributions

HY, XZ, and JL designed this study. JL and QD conducted the data analysis and wrote the manuscript. GL, ZH, and DZ carried out the field experiments. HZ helped data analysis. XW, CJ, XL, ZZ, and SW revised the manuscript. All authors contributed to the article and approved the submitted version.
